# Evaluating InPACT Intervention‐Context Fit in Resource‐Limited School Districts in Central Michigan

**DOI:** 10.1111/josh.70042

**Published:** 2025-07-07

**Authors:** Rebecca E. Hasson, Michele Marenus, Amy Wassmann, Andria B. Eisman, Thomas Templin, Lexie R. Beemer, Tiwaloluwa A. Ajibewa, Anna Schwartz, Lynn Malinoff, Ronald F. Zernicke

**Affiliations:** ^1^ School of Kinesiology University of Michigan Ann Arbor Michigan USA; ^2^ Saginaw Intermediate School District Saginaw Michigan USA; ^3^ Center for Health and Community Impact Wayne State University College of Education Detroit Michigan USA; ^4^ Institute for the Study of Children, Families, and Communities Eastern Michigan University Ypsilanti Michigan USA; ^5^ Department of Orthopedic Surgery and Biomedical Engineering University of Michigan Ann Arbor Michigan USA

**Keywords:** COVID‐19 pandemic, EPIS framework, hexagon discussion and analysis tool, physical activity intervention

## Abstract

**Background:**

Many school‐based physical activity (PA) interventions are implemented without adequately assessing their contextual fit, which can hinder their effectiveness and sustainment, ultimately reducing their impact on student health and well‐being. This study aimed to assess the pre‐implementation fit of the Interrupting Prolonged Sitting with ACTivity (InPACT) classroom PA intervention in one resource‐limited intermediate school district (ISD; encompassing 16 local districts and 32 elementary schools) in central Michigan.

**Methods:**

Assessments were conducted by the regional school health coordinator and ISD support staff during 2020–2021, using the Hexagon Discussion and Analysis Tool. Ratings on a 5‐point Likert scale were totaled for need, fit, support, evidence, usability, and capacity.

**Results:**

The first assessment score was 19/30; the second assessment score was 28/30, indicating a 47% increase in fit over a year. Field notes revealed a growing need for school‐based PA due to limited opportunities during COVID‐19. Improvements in support, fit, evidence, usability, and capacity were linked to enhanced knowledge and capacity of the regional school health coordinator.

**Implications for School Health Policy, Practice, and Equity:**

The initial misalignment between intervention and context highlights the importance of addressing contextual barriers in resource‐limited schools to effectively promote PA equity.

**Conclusions:**

By conducting multiple assessments, schools can identify and address barriers to intervention fit, increasing readiness and the likelihood of successful implementation.

## 1 Introduction

The *U.S. Physical Activity (PA) Guidelines for Americans* recommend that children and adolescents aged 6–17 engage in at least 60 min of moderate‐to‐vigorous PA daily to support overall health and well‐being [1]. However, national surveillance data show that most children fall short of these recommendations, increasing their risk for obesity and related chronic conditions [2]. Schools provide a vital setting to help close this PA gap [3, 4]. Among school‐based strategies, classroom‐based PA interventions have emerged as effective and evidence‐based approaches to increase students' daily activity levels [5, 6], with demonstrated benefits for cognitive functioning, cardiometabolic health, on‐task behavior, and academic performance [6, 7, 8]. Despite their potential, the implementation fidelity, and thus effectiveness of these interventions can vary due to the complex and dynamic nature of the school context [9, 10].

Classroom‐based PA interventions often face significant implementation challenges, particularly in resource‐limited school settings [11]. While adoption rates may be high, actual implementation frequently falls short [9, 12, 13, 14]. This gap is often due to insufficient consideration of the fit between the intervention and the school's specific context before adoption [15, 16, 17]. External pressures—such as policy mandates, funding opportunities, and perceived benefits—can lead schools to adopt multiple interventions rapidly, sometimes without fully assessing their capacity or aligning new programs with existing initiatives [18, 19, 20]. This can result in implementation fatigue among teachers and staff [17, 21, 22]. Conversely, other schools may avoid adopting any interventions altogether, leaving educators without access to effective strategies to support student health and academic success [22, 23, 24, 25]. Both scenarios underscore the importance of assessing intervention‐context fit prior to implementation.

Pre‐implementation efforts are essential to prepare schools for successful adoption of new interventions [17, 23, 26]. This involves identifying contextual factors, adapting interventions to fit the school context, and addressing potential barriers in advance [17, 23]. Without this foundational work, interventions are more likely to fail and less likely to produce meaningful public health outcomes [17, 23].

Implementation science offers tools to guide the pre‐implementation process. For example, the Exploration, Preparation, Implementation, and Sustainment (EPIS) framework [27] is a widely used multi‐phase framework designed to improve the implementation of evidence‐based practices. More specifically, EPIS can help assess the organizational structure and individual adopter characteristics that influence the fit between an intervention and the implementation context. As a multi‐phase implementation framework, EPIS consists of four phases: exploration (identifying implementer and organizational needs and aligning them with evidence‐based practices), preparation (assessing potential barriers and facilitators and developing supports to address them), implementation (monitoring progress), and sustainment (establishing accountability measures to promote long‐term success). The exploration and preparation phases—together known as pre‐implementation—are particularly critical to school contexts to ensure intervention‐context fit. EPIS has been successfully applied in various educational and health settings. For example, it has guided the implementation of Motivational Interviewing interventions in adolescent HIV care [28] and has been highlighted in systematic reviews for its broad applicability in school‐based initiatives [29].

This study focused on conducting pre‐implementation assessments of intervention‐context fit for Interrupting Prolonged sitting with ACTivity (InPACT), an evidence‐based classroom PA intervention in one resource‐limited intermediate school district (ISD) in central Michigan. The goal was to identify barriers and facilitators that influence implementation success in such settings. We hypothesized that systematically assessing contextual factors prior to implementation would lead to alignment between the intervention and school needs, thereby reducing barriers and promoting fidelity. Understanding the contextual landscape allowed us to identify specific areas for strategy development and address gaps that could hinder successful implementation.

## 2 Methods

### 2.1 Intervention

InPACT is an evidence‐based classroom PA intervention designed for resource‐limited schools [13, 30]. This manualized intervention incorporates five 4‐min activity breaks throughout the school day, providing students with up to 20 min of in‐class PA. To support high‐fidelity implementation, InPACT offers tailored intervention materials, teacher training, and technical assistance [30]. The intervention has successfully improved several student‐based outcomes, including increased student PA levels, improved on‐task behavior, enhanced enjoyment of PA, and greater goal attainment [13, 31]. It has also helped reduce disparities in classroom PA participation related to gender and asthma [13, 32].

To support long‐term sustainment, InPACT includes a train‐the‐trainer component [33]. Regional school health coordinators—representing 24 sites across Michigan—are trained in using the four‐phase EPIS framework, with key activities for each phase detailed in the InPACT implementation guide. Utilizing this guide, these coordinators deliver training and technical assistance to school leaders to ensure consistent implementation statewide.

### 2.2 Context

This study was conducted in one resource‐limited ISD, encompassing 16 local districts and 32 elementary schools, serving 11,830 children—70% of whom qualify for free and reduced lunch [34]. In Michigan, ISDs function as regional education service agencies that provide support to local school districts overseeing individual schools.

Prior to the pre‐implementation assessments, the ISD faced several challenges to PA promotion. District wellness policies did not include classroom PA breaks [35], and only 7 out of 32 elementary schools mentioned health in their mission statements (unpublished data). Additionally, the limited number of physical education teachers (16 across all 32 schools) meant that teachers were often required to travel between schools, impeding consistent PA delivery. The COVID‐19 pandemic further exacerbated these challenges by reducing in‐person instruction and limiting students' PA opportunities [36].

### 2.3 Instrumentation

The Hexagon Discussion and Analysis Tool (Hexagon Tool) [37] was used to assess the contextual fit and feasibility of implementing InPACT in the ISD. Aligned with the Exploration phase of the EPIS framework, the tool evaluates six key indicators essential for implementation success, including outer and inner context considerations [27]. Outer context considerations refer to external factors such as policy and funding landscapes, sociopolitical influences, state or district regulations, and broader community or stakeholder priorities that impact whether and how an intervention is adopted. Inner context considerations focus on organizational and individual‐level factors, including leadership support, staff capacity, organizational culture, available resources, and the attitudes and readiness of those expected to implement the intervention. By exploring these factors, the Hexagon Tool provides guidance on factors that can hinder or enable implementation within the contexts where they are applied.

The tool facilitated structured discussions with key school partners, including ISD staff and school principals, to assess the perceived fit and feasibility of adopting InPACT in select schools. The six indicators were: *evidence*, *supports*, *usability*, *need*, *fit*, and *capacity* (see Table 1). These are grouped into two categories: *intervention indicators* (focusing on the intervention itself) and *implementing site indicators* (addressing the context in which the intervention will be implemented).

**TABLE 1:** Hexagon tool questions adapted for InPACT.

Indicator	Description of indicator	Original question	Adapted questions
Need	Identification of focus population and subpopulations Use of multiple data sources and disaggregated data to understand needs and assets Community perception of needs and assets	Who is the identified focus population? Are there subpopulations? If so, please describe. What is/are the identified needs of these population(s)? What are the root causes of these needs? What are the identified assets of these population(s)? Was an analysis of data conducted to identify specific area(s) of need relevant to the program or practice? If yes, what data were analyzed? Were these data disaggregated by race, ethnicity, language, and other characteristics specific to the focus population and subpopulation(s)? How do members of the focus population perceive their need? What do they believe will be helpful? How were community members engaged to assess perception of need? If the program or practice is implemented, what could potentially change for these population(s)?	What population would benefit from InPACT? What are the needs of this population? Was an analysis conducted to identify areas of need relevant to InPACT? What are the parents and community perceptions of the need for InPACT? Does InPACT address the system's needs and gaps?
Fit	Fit with community values, culture and history Impact on other initiatives Alignment with other priorities of the implementing site	How does the program or practice fit with priorities of the implementing site? How does the program or practice fit with family and community values and assets in the impacted community, including the values of racially, ethnically, culturally, and linguistically specific populations? What other initiatives currently being implemented will intersect with the program or practice? How does the program or practice fit with other existing initiatives? Will the other initiatives make it easier or more difficult to implement the proposed program or practice and achieve the desired outcomes? How does the program or practice fit with the community's history relevant to the identified need or focus population? How does it disrupt the community's history or systems? What is the potential impact of this fit or disruption?	How does InPACT fit with the priorities of your school? Does InPACT fit the family and community values, culture, and history? Are there any other initiatives in your school that will intersect with InPACT? How does the intervention fit with those initiatives? How does InPACT align with the school's organizational structure?
Supports	Expert assistance External resources for implementing sites	Is there a qualified “expert” (e.g., consultant, program developer, intermediary, technical assistance provider) who can help with implementation over time? If yes, list names and/or organization (e.g., Center, University) and contacts. Are there start‐up costs for implementation of the program or practice (e.g., fees to the program developer)? If yes, itemize in notes section. What does the implementing site receive for these costs? Are there curricula and/or other resources related to the program or practice readily available? If so, list publisher or links. Are the materials representative of the focus population who will be receiving and delivering the program or practice? What is the cost of these materials? Enter in notes section. Is training and professional development related to the program or practice readily available? Is training culturally sensitive? Does the training use adult learning best practices? Does it address issues of race equity, cultural responsiveness or implicit bias? Include the source of training and professional development. What is the cost of this training? Enter in notes section. Is coaching available for the program or practice? Is coaching culturally sensitive? If so, list coaching resources and cost in notes section. Are sample job descriptions and interview protocols available for hiring or selecting new staff for the program or practice? Have these job descriptions and protocols been run through a racial equity impact analysis? If so, identify here and list any costs associated. Is guidance on administrative policies and procedures available, such as what changes to existing processes will be needed? Have recommended policies and procedures been run through a racial equity impact analysis? If so, identify resources and any costs associated. Are there resources available to develop a data management plan for the program or practice (including data system and monitoring tools)? If so, identify resources and any costs associated. Is there a recommended orientation to facilitate buy‐in for staff, key stakeholders and collaborative partners? If so, explain/describe briefly in notes section.	Is there a qualified “expert” who can help implement InPACT over time? Are there start‐up costs for implementing InPACT? Are there curricula and other resources related to InPACT readily available? What do they cost? Is the training and professional development for InPACT readily available? Does it address issues of race equity, cultural responsiveness, or implicit bias? Is coaching available for InPACT? Is there guidance on administrative policies and procedures for InPACT? Are there resources to develop a data management plan for InPACT? Is there a recommended InPACT orientation to facilitate “buy‐in” for staff, key stakeholders, and collaborative partners?
Evidence	Outcome, fidelity, and cost effectiveness data Strength of evidence: for whom and in what conditions	Are there research data available to demonstrate the effectiveness (e.g., randomized trials, quasi‐experimental designs) of the program or practice? If yes, provide citations or links to reports or publications. What is the strength of the evidence? Under what conditions was the evidence developed? What outcomes are expected when the program or practice is implemented as intended? How much of a change can be expected? If research data are not available, are there evaluation data to indicate effectiveness (e.g., pre/post data, testing results, action research)? If yes, provide citations or links to evaluation reports. Is there practice‐based evidence or community‐defined evidence to indicate effectiveness? If yes, provide citations or links. Is there a well‐developed theory of change or logic model that demonstrates how the program or practice is expected to contribute to short‐term and long‐term outcomes? If yes, provide citations or links. Do the studies (research and/or evaluation) provide data specific to the setting in which it will be implemented (e.g., the program or practice been researched or evaluated in a similar context)? If yes, provide citations or links to evaluation reports. Do the studies (research and/or evaluation) provide data specific to effectiveness for racially, ethnically, culturally and linguistically specific populations? If yes, provide citations or links specific to effectiveness for families or communities from diverse cultural groups.	Are there research data to demonstrate that InPACT is effective? How strong is the evidence of effectiveness? Under what conditions? What are the expected outcomes of InPACT when it is implemented as intended? How much of a change can be expected? Has InPACT been researched in a similar context to where you will implement? Is it effective in diverse culture groups?
Usability	Well‐defined program Adaptations for context and populations	Is the program or practice clearly defined (e.g., what it is, for whom it is intended)? Are core features of the program or practice identified, listed, named (e.g., key components of the program or practice that are required in order to be effective)? Is each core feature well operationalized (e.g., staff know what to do and say, how to prepare, how to assess progress)? Is there guidance on core features that can be modified or adapted to increase contextual fit? Do these core features differ for specific populations, such as for racial/ethnic groups? If so, how? Is there a fidelity assessment that measures practitioner behavior (i.e., assessment of whether staff use the practice as intended)? If yes, provide citations, documents, or links to fidelity assessment information. Has the program or practice been adapted for use within racially, ethnically, culturally, and linguistically specific populations and/or is there a recommended process for gathering input from the focus population and community on culturally specific enhancements? What do we know about the key reasons for previous successful replications? What do we know about the key problems that led to unsuccessful replication efforts previously? Are there mature sites with successful histories of implementing the program or practice who are willing to be observed?	Is InPACT clearly defined? Are the core features identified, listed, named? Is there guidance on how to modify or adapt InPACT to increase contextual fit? What do we know about the key reasons for previous InPACT successful replications? What do we know about the key problems? Are there mature sites with successful histories of implementing InPACT who are willing to be observed? Are there fidelity assessments that measure practitioner behavior of InPACT implementation?
Capacity	Implementation costs Resources needed and available for implementation	Typically, how much does it cost to run the program or practice each year? Are there resources to support this cost? If the current budget cannot support implementation, outline a resource development strategy. What are the staffing requirements for the program or practice (number and type of staff, e.g., education, credentials, content knowledge, cultural competency, and cultural congruency)? Does the implementing site currently employ or have access to staff that meet these requirements? If so, do those staff have a cultural and language match with the population they serve, as well as relationships in the community? What administrative practices must be developed or refined to support the use of this program or practice? Is leadership knowledgeable about and in support of this program or practice? Do leaders have the diverse skills and perspectives representative of the focus population? Do staff have the capacity to collect and use data to inform ongoing monitoring and improvement of the program or practice? What administrative policies or procedures must be adjusted to support the work of practitioners and others to implement the program or practice? Will the current communication system facilitate effective internal and external communication with stakeholders, including the focus population? Will the program or practice require use of or changes to building facilities? Use notes section to explain. List required uses of and/or changes. Include costs if known. Does the program or practice require new technology (hardware or software, such as a data system)? Use notes section to explain. List required hardware and/or software. Include costs if known. Does the program or practice require use of or changes to the monitoring and reporting system? Use notes section to explain. List required uses of and/or changes. Include costs if known.	What are the staffing requirements for implementing InPACT? Does your school employ or have access to staff that meet these requirements? What administrative practices must be developed or refined to support the use of InPACT? Does the staff have the capacity to collect and use data to inform ongoing monitoring and improvement of InPACT? Will the current communication system facilitate effective internal and external communication with stakeholders, including impacted families and the community? Is your school able to sustain the staff, coaching, data systems, performance assessment, and administration needed to implement InPACT? Does your school have the financial, structural, and cultural responsibility capacity to implement InPACT? Is the buy‐in process operationalized for practitioners and families?

The intervention indicators consist of *evidence*, *supports*, and *usability*. *Evidence* assesses research outcomes, implementation fidelity, cost‐effectiveness, and the strength of evidence (e.g., for whom and under what conditions the intervention is effective [38, 39]). *Supports* address available technical assistance and external resources [39]. *Usability* examines how clearly the intervention is defined and whether core components can be adapted to specific settings [39].

The implementing site indicators are *need*, *fit*, and *capacity*. *Need* captures the perceived necessity of the intervention by the target population [39]. *Fit* evaluates alignment with community values, culture, and institutional priorities [39]. Finally, *capacity* considers the costs and resources required for implementation [39].

Each indicator is accompanied by discussion questions to guide evaluation. At the end of each section, participants rated the indicator using a 5‐point Likert scale; higher scores indicate stronger fit. A full list of questions is available at: https://nirn.fpg.unc.edu. The Hexagon Tool has been widely applied across various fields, including education and mental health programs, where educators and early childhood specialists have used it to assess program alignment with their unique environments and ensure interventions meet student needs [37, 40].

### 2.4 Procedures

Two Hexagon Tool assessments were conducted to gather longitudinal data on contextual fit: one in March 2020 (pre COVID‐19 pandemic) and the second in February 2021 (during the pandemic). The first assessment occurred just before COVID‐19‐related school closures, while the second followed a period of disrupted instruction (i.e., virtual learning). Both assessments considered the shifting needs and realities introduced by the pandemic. Data collection was conducted by the regional school health coordinator (A.W.), who had prior experience using the tool and facilitating group discussions. The InPACT research team did not participate in data collection.

#### 2.4.1 First Assessment

The first assessment was conducted as a single session evaluating InPACT's fit across the ISD. The session was led by the regional school health coordinator and included five additional participants representing both inner (school) and outer (regional) contexts: the Director of Instructional Services, Executive Director of Instruction, Lead Nutrition Facilitator, and two elementary principals.

As the facilitator, the coordinator selected and adapted discussion questions to suit InPACT and assembled relevant statistics in advance (see Table 1). The session lasted approximately 2 h. To maximize efficiency, the coordinator completed several items related to the intervention indicators beforehand (e.g., evidence, supports, and usability), allowing the group to focus on the implementing site indicators during the discussion. Given the impending shift to remote learning, participants reflected on the feasibility of implementing InPACT under uncertain and evolving conditions.

#### 2.4.2 Second Assessment

The second assessment followed the same structure and duration (2 h), led again by the regional school health coordinator and involving two ISD nutrition facilitators. School‐level staff were unable to attend due to scheduling conflicts. To ensure independent ratings, participants did not review notes or scores from the first session. During this discussion, the team acknowledged pandemic‐related shifts in instructional delivery and adopted a more flexible view of implementation feasibility in the context of hybrid and remote learning.

### 2.5 Scoring

Each participant rated the six indicators individually before sharing with the group. Discrepancies in scoring were resolved through group discussion until consensus was reached. A dialogic communication approach was used to encourage inclusive, respectful dialogue and ensure diverse perspectives were considered. The aim was to foster discussions and ultimately reach consensus on scoring.

The coordinator recorded notes summarizing each discussion, including major concerns and rationale behind the scores. These qualitative insights were documented alongside the numerical ratings. Final scores were calculated by summing the six indicator scores. The same procedures were applied for both assessments. Importantly, ratings were not the sole basis for adoption decisions; final recommendations also incorporated discussion notes.

### 2.6 Data Analysis

Both quantitative ratings and qualitative field notes were shared with the InPACT research team for analysis. One team member (M.W.M) conducted a descriptive thematic analysis of the field notes, which took approximately 10 h [41]. Three additional team members (L.B., A.S., T.A.) reviewed and verified the themes to ensure alignment with the raw data, contributing 2–3 h each. To further validate the findings, the resulting themes were shared with the regional school health coordinator for review. This member‐checking step ensured accurate representation of discussions and decisions. Field notes are included in Supporting Information S1.

## 3 Results

Table 2 presents Hexagon Tool results assessing the intervention‐context fit of InPACT within the ISD. A summarized overview of scores along with a descriptive analysis for each indicator is presented below.

**TABLE 2:** Data display table.

Indicator	2020		2021	
	Score	Field notes	Score	Field notes
Need	3	Physical education (PE) time cut at certain schools. Students are sedentary at home. InPACT would help reach 60 min of activity. Indoor recess has minimal physical activity (PA). Current practices lack resources, training, and effectiveness data. Questions about school wide implementation.	5	Targeting elementary schools in the county. PE has either been eliminated or reduced due to COVID‐19 as well as sports and movement during the day. Schools face‐to‐face, virtual, or hybrid Obesity rates are estimated to rise. Staff are noticing students need to move more and want resources and support for physical activity. Michigan is 5th worst state in the US in obesity rates. Data from Michigan School Health Survey System show that 48.6% of 7th graders state they are trying to lose weight, large amounts of screen time. PA drops by 75% from ages 9 to 15. InPACT in combination with PE and recess addresses those needs.
Fit	3	Desire for school‐wide and community‐wide involvement. Question about when to implement. Desire for it not to be an add‐on, not just an additional burden. Fear of being methodical; brain breaks are a place for autonomy. Fit with Whole Child and Health Body Healthy Mind (HBHM) curriculum. Questions about alignment, potential reallocation of time, training, and cost. Time audit data may be helpful.	4	Aligns well with Healthy Bodies, Healthy Minds (HBHM). Links with brain breaks. Community Health Improvement Plan Steering Committee to help reduce obesity rates. Helps meet Center for Disease Control and Prevention physical activity guidelines. Parents are not always active; this will give students an opportunity to be active at school. Schools are already implementing activity breaks using GoNoodle/Just Dance. Part of Michigan Model for Health training. Concerns from first assessment remain.
Supports	3	Questions about stipend for train‐the‐trainer program, teacher buy‐in, data collection, and autonomy. Concern that PhD staff at University of Michigan (UM) not understanding the K‐12 dynamics Flexibility is a plus Questions about what the training looks like, differences with 31n school‐based mental health services, and staff at UM	5	UM staff ISD's Whole Child Program Director HBHM staff Training for teachers and training for trainers Michigan Health Endowment funds and additional funds to improve movement at school. Buy‐in from administration, PE teacher, classroom teachers, 31n staff.
Evidence	4	Certain schools within the ISD do not have a comparable program. Pilot phase for evidence. Questions about data on discipline, attendance, academic improvements. Start small.	5	Strong evidence InPACT provides up to 20 min of daily PA, high student participation, enjoyment, and on‐task behavior in the classroom. Previously implemented in similar districts throughout the state. Interested in data on discipline, attendance, and academic improvement.
Usability	3	Pilot phase Part of first replication Diverse setting of ISD	5	Intervention well‐defined with replication in similar districts. Concern about adding to teacher's workload. COVID‐19 barrier. InPACT intervention allows for adaptions and flexibility.
Capacity	3	No cost Potential to implement at schools not using HBHM curriculum. Ability to expand because of train‐the‐trainer program. One trainer per building—can be PE teachers, social workers, core team as ISD, behavior interventionist.	4	Whole Child Program director already trained and can train classroom teachers. Core county team will be trained to assist Whole child program director (in future). Does not require ongoing costs for implementation, just time. Districts can continue with evaluation once grant ends.
Total	19/30		28/30	

### 3.1 First Assessment‐March 2020

The first pre‐implementation assessment was conducted in early March 2020, just one day before schools in Michigan were closed due to the COVID‐19 pandemic. As such, the findings from this assessment reflect conditions prior to the onset of the pandemic.

#### 3.1.1 Need (3/5)

Moderate need was indicated. Qualitative results attributed this to reduced PE time, limited PA during indoor recess, and sedentary student behavior at home. InPACT was viewed as a promising solution to help students meet daily PA recommendations.

#### 3.1.2 Fit (3/5)

A moderate fit with existing initiatives was reported. This stemmed from concerns about professional development schedules and potential workload increases for teachers. Field notes underscored the importance of framing InPACT as aligned with current routines rather than an added burden.

#### 3.1.3 Support (3/5)

A moderate level of support emerged. Field notes documented questions about training stipends, implementation structure, and strategies to generate buy‐in from teachers. School staff emphasized the importance of flexibility and understanding of the K‐12 context by intervention developers.

#### 3.1.4 Evidence (4/5)

Participants acknowledged moderate‐to‐high evidence recognizing that InPACT was still in the pilot phase. There was some uncertainty about its impact on broader outcomes such as discipline, attendance, and academic performance. Despite this, school staff viewed the program as evidence‐informed, citing published research showing that classrooms can achieve two‐thirds of the recommended daily school‐based PA (20 min) and that 99% of students return to task after an activity break [13, 30]. These outcomes contributed to a higher evidence rating relative to other domains.

#### 3.1.5 Usability (3/5)

Moderate usability was reported. ISD and school staff highlighted that InPACT was still undergoing refinement and noted their ISD would be among the first to replicate the program –an opportunity viewed with cautious optimism.

#### 3.1.6 Capacity (3/5)

Moderate capacity was identified. No financial costs were associated with implementation, and there was potential for broader adoption through the train‐the‐trainer model. Field notes also mentioned that InPACT could fill gaps in schools lacking wellness programming.

### 3.2 Second Assessment‐February 2021

Following the initial assessment, the COVID‐19 pandemic restricted the ISD's ability to move forward with the adoption and implementation of the program. During the intervening period, the ISD worked to develop supports and sustain readiness for future implementation. A second assessment was conducted in February 2021 to reassess contextual fit and capacity in light of the pandemic's ongoing impacts on school contexts.

#### 3.2.1 Need (5/5)

Highest need was reported. This two‐point increase reflected the impact of COVID‐19 on children's sedentary behavior and the reduction in school‐based PA. The isolation and reduced PA during remote learning made the case for in‐class activity breaks even more compelling. ISD staff strongly reaffirmed InPACT's relevance, especially for helping students reengage in movement post‐pandemic.

#### 3.2.2 Fit (4/5)

Moderate‐to‐high fit was recognized. This improvement was attributed to a better understanding of how InPACT aligned with ongoing programs such as *Healthy Bodies, Healthy Minds*, which used Fitbits and short PA breaks. Field notes also cited alignment with *Brain Breaks*, Community Health Improvement Plan (CHIP) Steering Committee goals, and existing classroom‐based PA resources like *GoNoodle* and *Just Dance*. ISD staff also noted that InPACT could be integrated into the *Michigan Model for Health* (a comprehensive universal health education curriculum) training [33, 42]. The fit of InPACT with these programs became even more apparent after the pandemic as schools recognized the value of structured breaks in addressing both the PA and mental health needs of students.

#### 3.2.3 Support (5/5)

Highest level of support was documented. Field notes listed broad “buy‐in” from ISD leadership, district and school administrators, classroom teachers, physical education staff, and mental health professionals (see Supporting Information S1). Support was bolstered by increased familiarity with available resources and intervention developer engagement. Again, the pandemic heightened the recognition of the importance of such interventions, and the buy‐in from various stakeholders reflected this urgency.

#### 3.2.4 Evidence (5/5)

A one‐point increase in perceived evidence‐with the highest level of evidence documented‐was attributed to growing awareness of peer‐reviewed publications demonstrating InPACT's effectiveness in similar school settings [30, 31, 32]. The COVID‐19 pandemic underscored the importance of having strong evidence to support PA interventions, especially in uncertain times when schools were more cautious about implementing new initiatives.

#### 3.2.5 Usability (5/5)

ISD staff recognized highest usability, citing improved understanding of intervention components and confidence in adapting InPACT across diverse school contexts. While COVID‐19 restrictions were still a barrier, flexibility in delivery was highly valued.

#### 3.2.6 Capacity (4/5)

Moderate‐to‐high capacity was reported. This increase reflected greater clarity around the train‐the‐trainer structure and implementation roles within the ISD. Moreover, during the pandemic, additional attention was given to how capacity could be built virtually, allowing for the continued training of staff even with the challenges from COVID‐19.

In summary, results from the two assessments show an upward trend in perceived fit, feasibility, and impact of InPACT across all indicators. Qualitative feedback revealed growing familiarity, confidence, and enthusiasm for the intervention over time, suggesting a strong foundation for sustained implementation.

## 4 Discussion

This study provides new insight into how pre‐implementation assessments can support successful adoption of classroom‐based PA interventions in resource‐limited school contexts. Specifically, it highlights (1) how contextual shifts—particularly the COVID‐19 pandemic—affected intervention fit and feasibility; (2) how increases in stakeholder knowledge and capacity—enhanced readiness for implementation; and (3) the value of applying implementation science frameworks, such as EPIS, to guide and evaluate the process. The following discussion explores these themes in detail and offers practical implications for improving intervention fit, building implementation capacity, and supporting the long‐term sustainment of PA interventions in resource‐limited school systems.

### 4.1 Intervention‐Context Fit and Changing Conditions

Pre‐implementation assessments of readiness for evidence‐based interventions can help prevent future implementation failures [17, 23]. In this study, the initial Hexagon Tool assessment of InPACT within the ISD showed a moderate score (19 out of 30), which improved 47% over the following year, reaching 28 out of 30. Improvements were across all six indicators, largely influenced by the disruption of structured PA opportunities due to the COVID‐19 pandemic. The pandemic acted as a powerful external catalyst, reshaping the perceived need for interventions like InPACT.

Consistent with the EPIS framework, changes in both the outer and inner contexts were key determinants of evolving intervention‐context fit. The COVID‐19 pandemic, a major disruption to the outer context, significantly altered the school context [43]. In fall 2020, districts in the resource‐limited ISD chose between in‐person or virtual learning, with families also given the option to stay virtual. Many in‐person schools adopted A/B schedules, with students alternating days and all learning virtually on Fridays. Elementary students remained in self‐contained classrooms. Masks were optional in classrooms but required in hallways. To limit movement, special subjects, lunch, and PE took place in classrooms. PE was particularly limited due to concerns about virus transmission from heavy breathing, restricting high‐intensity activities. Remote learning further reduced access to PE, extracurriculars, and afterschool PA [36]. This shift in the socio‐political environment heightened the perceived urgency for school‐based PA interventions, even as feasibility became more complex due to safety protocols.

### 4.2 Building Readiness Through Capacity‐Building

A notable change between the two assessment periods was the increased knowledge and capacity of the regional school health coordinator—an EPIS inner context determinant. Over the course of a year, the coordinator had weekly meetings with the InPACT principal investigator, resulting in over 50 h of engagement. During this time, the coordinator completed the InPACT train‐the‐trainer program, which included three online trainings (9 h of professional development) focused on implementing InPACT in classrooms [33]. The coordinator was equipped with the scientific rationale, program objectives, core components, and risk management strategies necessary for safe implementation. For example, the coordinator was trained to address health concerns such as asthma or type 1 diabetes during activity breaks and to integrate breaks into classroom routines. These capacity‐building activities reflect EPIS preparation phase strategies designed to align individual adopter characteristics with intervention demands.

### 4.3 Partnership and Systems‐Level Change

The health coordinator also collaborated with the InPACT research team on district‐wide PA policy assessments, staff interviews, and the creation of an implementation guide [33, 35]. These collaborative efforts were critical for aligning intervention delivery with school priorities and boosting readiness across multiple levels of the system. As a result, scores on the Hexagon Tool improved in all six domains: *support*, *fit*, *evidence*, *usability*, *capacity*, and *need*. Findings underscore the value of investing in trusted school partners and using implementation science frameworks to build the conditions necessary for successful and sustained adoption of evidence‐based practices.

### 4.4 Implications for Practice and Sustainment

Results from the two assessments revealed a consistent increase in readiness and alignment with ISD needs and priorities. ISD and school staff reported a stronger perceived need and broader support for InPACT during the second assessment, indicating the growing relevance of PA interventions as schools recovered from pandemic‐related disruptions.

Key strategies that contributed to improved readiness and sustainment included the use of a train‐the‐trainer model, flexibility in intervention delivery, and alignment with statewide health curricula [33, 42]. These strategies illustrate practical ways to embed scalable PA interventions into daily classroom routines and enhance buy‐in from educators and administrators. For example, the train‐the‐trainer model has been shown to build local capacity and increase fidelity of cognitive behavioral therapy interventions delivered in school settings [44]. Flexibility in intervention delivery—such as allowing teachers to choose when and how to implement PA breaks—has been found to increase intervention uptake, especially in schools with limited time or competing demands [30]. Aligning PA interventions with existing health curricula or academic goals further increases their perceived value and feasibility. In Michigan, for instance, integrating InPACT resources with the *Michigan Model for Health* curriculum helped streamline implementation by embedding PA within an already‐required instructional framework [42].

Prior research supports these findings—when interventions are perceived as compatible with existing systems, aligned with teachers' instructional goals, and supported by leadership, they are more likely to be adopted with fidelity and sustained over time. Durlak and DuPre identified organizational fit and principal support as key factors influencing implementation quality and sustainment [17]. Similarly, Domitrovich et al. emphasized the importance of aligning new interventions with a school's culture, structure, and existing priorities to reduce implementation resistance [16]. Together, these studies underscore the critical role of contextual alignment and organizational support in ensuring the long‐term success of school‐based interventions.

### 4.5 Limitations

Several limitations of the current study should be acknowledged. First, the Hexagon Tool discussions were not audio‐recorded, limiting the ability to conduct a full thematic content analysis. However, rigorous field notes allowed for a descriptive content analysis. Second, a validated measure of intervention‐context fit was not used; instead, the Hexagon Tool—a pragmatic, evidence‐informed instrument selected by the partnering ISD—was employed to ensure comparability of results with other initiatives in the ISD. Third, fewer school‐level team members were present for the second assessment due to scheduling conflicts, including the absence of a key partner—the principals. This may have partially influenced the higher scores observed. Despite these limitations, the longitudinal design allowed for the evaluation of fit and readiness over time in a low‐resource school context. Notably, the ISD—and one of the elementary school principals involved in the initial assessment—chose to adopt and pilot the InPACT intervention [35].

## 5 Conclusions

This study provides evidence that repeated assessments of intervention‐context fit can guide strategic adaptations and support readiness over time. While findings are specific to InPACT and one ISD, the process of assessing and improving fit is broadly applicable to other settings and interventions [37]. Additionally, using a multi‐phase implementation science framework like EPIS can help schools proactively identify and address barriers to implementation, thereby narrowing the gap between adoption and high‐fidelity, sustained delivery of evidence‐based classroom PA interventions.

## Ethics Statement

This study was considered Exempt Human Research by the Institutional Review Board (HUM00188402) due to the following reason: “research conducted in established or commonly accepted educational settings, involving normal educational practice, such as: (i) research on regular and special education instructional strategies, or (ii) research on the effectiveness of or the comparison among instructional techniques, curricula, or classroom management methods.”

## Conflicts of Interest

The authors declare no conflicts of interest.

## Supporting information


**Data S1.** Supporting Information.

## Data Availability

The data that supports the findings of this study are available in the supplementary material of this article.
