# Supplementary material for: Evaluating InPACT Intervention‐Context Fit in Resource‐Limited School Districts in Central Michigan
Source: J Sch Health. 2025 Jul 7;95(9):699–709. doi: 10.1111/josh.70042 (PMC12315085; doi:10.1111/josh.70042)
Supplement: Supplementary file 1 — Data S1. Supporting Information. [file JOSH-95-699-s001.pdf]

The Hexagon Tool: An Exploration Tool **Please create a copy and rename.**
Resource: University of North Carolina
Notes Page

**Project Name**	**InPACT at School**
**Team Members**	**5 members at the ISD**
**Date**	**March 11, 2020**
**Score (Individual scores after titles of columns)**	**19/30**

**NEED (3)**<br>(page 7)<br>Notes Page	**FIT (3)**<br>(page 8)<br>Notes Page	**SUPPORTS (3)**<br>(page 5)<br>Notes Page	**EVIDENCE (4)**<br>(page 4)<br>Notes Page	**USABILITY (3)**<br>(page 6)<br>Notes Page	**CAPACITY (3)**<br>(page 9/10)<br>Notes Page
**Need in school, district, state**<br>● Target population identified<br>● Disaggregated data indicating population needs<br>● Parent & community perceptions of need<br>● Addresses service or system gaps	**Fit with current initiatives**<br>● Alignment with community, regional, state priorities<br>● Fit with family and community values, culture and history<br>● Impact on other interventions & initiatives<br>● Alignment with organizational structure	**Resources and supports**<br>● Expert assistance<br>● Staffing<br>● Training<br>● Coaching and supervision<br>● Racial equity impact assessment<br>● Data systems technology supports<br>● Administration and system	**Evidence**<br>● Strength of evidence--for whom in what conditions<br>○ Number of studies<br>○ Population similarities<br>○ Diverse culture groups<br>○ Efficacy or effectiveness<br><br>● Fidelity data<br>● Cost -- effectiveness data<br>● Outcomes -- is it worth it?	**Readiness for replication**<br>● Well-defined program<br>● Mature sites to observe<br>● Several replications<br>● Adaptations for context	**Capacity to implement**<br>● Staff meet minimum qualifications<br>● Able to susten staff, coaching, training, data systems, performance assessment, and administration<br>○ Financial capacity<br>○ Structural capacity<br>○ Cultural responsivity capacity<br>● Buy-in process operationalized<br>○ Practitioners<br>○ Families
**A lot of our schools have had their PE time cut**<br>**Students at home are sedentary time - screen time - Help reach the 60 minutes of activity;**<br>**Indoor recess has minimal physical activity**<br><br>**There are things in place- brain breaks but variety of resources without cohesive decision around resource, training, effectiveness data, or necessarily attention to get to recommended daily dose of physical activity**	**Needs to be school wide and whole community**<br><br>**Time of year? Right now or next year? - Not this year per AW**<br>**UofM wants to be very intentional.**<br><br>**Teachers are in the process of simplifying Tier I instruction - would that be part of the training? PD District is already set. Sub costs? Release Time?**<br><br>**We don't want this to be just one more thing. Not an add on.**	**Is there a stipend for the TOT?**<br>**What does the training look like?**<br>**Buy-in from teachers is a concern?**<br>**Data? - U of M will also have an evaluator. Districts would need to collect the data.**<br><br>**It would be embedded. Are there a large variety of strategies for autonomy? Yes. How scripted is this?**<br><br>**Is the training more of the resources or is it training them on how to use the strategies?**	**BR does not have a comparable program right now. Nor does Chesaning**<br><br>**2/3rd of recommended activity**<br>**30 on task rate - 90%**<br><br>**Still in piloting phases.**<br>**Have they been collecting discipline, attendance, academic improvements?**<br><br>**Comparable data - start small in a building?**	**Pilot phase.**<br>**We are being offered to be part of the first replication.**<br><br>**Diverse settingings**<br>**Rural, Urban, Socio-Economic Status, Ethnicity**	**No cost to the school or ISD.**<br><br>**May need to look at non-HBHM schools and an eclectic mix**<br><br>**We would have the ability to expand because of TOT.**<br><br>**Who would the TOTs be?**<br>**PE Teachers**<br>**GE Social Workers**<br>**Core Team at ISD**<br>**One person per building**<br>**Behavior Interventionist**<br>**MPH would be offered so they have an understanding.**

The Hexagon Tool: An Exploration Tool **Please create a copy and rename.**
Resource: University of North Carolina
Notes Page

**Big Rock has a MHP that is tying it in.<br>Would it be schoolwide?**	**Fear of being methodical - Brain breaks are a place for autonomy.<br>Fit with whole child - there are a lot of initiatives going on already.<br>PE is not offered daily.<br>Is it aligned enough to be helpful. How might we need to reallocate time. Time audit data may be helpful. 20 minutes total<br>Fit with HBHM - extension**	**Not a 100% sure what this training is going to look like. There are video resources on the website for students and staff. What are the differences between this and 31n supports? Who are the staff at UofM?<br><br>The concern would be that the PhD staff at UofM not understanding the K-12 dynamics<br><br>Nice in the flexibility and who the TOT will be.**			

The Hexagon Tool: An Exploration Tool **Please create a copy and rename.**
Resource: University of North Carolina
Notes Page

Project Name	InPact at School
Team Members	3 team members from ISD
Date	February 24, 2021
Score	28/30

NEED (5)<br>(page 7)<br>Notes Page	FIT (4)<br>(page 8)<br>Notes Page	SUPPORTS (5)<br>(page 5)<br>Notes Page	EVIDENCE (5)<br>(page 4)<br>Notes Page	USABILITY (5)<br>(page 6)<br>Notes Page	CAPACITY (4)<br>(page 9/10)<br>Notes Page
**Need in school, district, state**<br>● Target population identified<br>● Disaggregated data indicating population needs<br>● Parent & community perceptions of need<br>● Addresses service or system gaps	**Fit with current initiatives**<br>● Alignment with community, regional, state priorities<br>● Fit with family and community values, culture and history<br>● Impact on other interventions & initiatives<br>● Alignment with organizational structure	**Resources and supports**<br>● Expert assistance<br>● Staffing<br>● Training<br>● Coaching and supervision<br>● Racial equity impact assessment<br>● Data systems technology supports<br>● Administration and system	**Evidence**<br>● Strength of evidence--for whom in what conditions<br>○ Number of studies<br>○ Population similarities<br>○ Diverse culture groups<br>○ Efficacy or effectiveness<br><br>● Fidelity data<br>● Cost -- effectiveness data<br>● Outcomes -- is it worth it?	**Readiness for replication**<br>● Well-defined program<br>● Mature sites to observe<br>● Several replications<br>● Adaptations for context	**Capacity to implement**<br>● Staff meet minimum qualifications<br>● Able to susten staff, coaching, training, data systems, performance assessment, and administration<br>○ Financial capacity<br>○ Structural capacity<br>○ Cultural responsivity capacity<br>● Buy-in process operationalized<br>○ Practitioners<br>○ Families
**Target Population:**<br>Elementary Schools in Saginaw County.<br><br>**Data indicating population needs and staff perceptions of need:**<br><br>COVID has altered the way education is implemented, with many schools starting the school year virtual. While some schools are back face to face, we still have many that are not. Also, hybrid schedules (2 days in person, 3 days virtual) limits the amount of time they are in class. Students	**Alignment:**<br><br>● Links well with Healthy Bodies, Healthy Minds (SNAP Ed work) programming as we include FitBits (3-8 min PA activities)<br>● Links with Brain Breaks teachers are implementing throughout the day<br>● CHIP Steering Cmte as we look to reduce obesity rates in Saginaw County<br>● CDC's recommendation of 60 minutes of PA a day for children<br>● Not all parents are physically	**Supports and Resources:**<br><br>● U of M Staff<br>● SISD's Whole Child Programs Director<br>● Healthy Bodies, Healthy Minds staff<br>● Training for Teachers<br>● Training for Trainers (sustainability/local experts): in the future<br>● U of M evaluation staff<br>● Michigan Health Endowment Fund grant<br>● Funds to support improvements to hallway and playground movement	**Evidence:**<br><br>In other schools, the InPACT program has:<br><br>● Added 20 min of extra PA<br>● 99% of children are back on task within 30 seconds of completing activity<br>● Children report high enjoyment of activity breaks (4.2/ 5)<br>● 78% of participating classrooms achieved their weekly participation goals<br><br>Schools that participated were in	**Readiness:**<br><br>InPACT at School is already a well defined program with several replications from districts in other Michigan counties that are similar to Saginaw ISD's student population.<br><br>The main issue is the barrier that COVID has played in getting things started. While buildings understand the need for movement, the one concern is "adding to the plates" of already stressed, overworked teachers.	**Capacity:**<br><br>Whole Child Program Director has been trained in InPACT at School and will provide training to classroom teachers in the buildings that participate.<br><br>FUTURE: A core county team will be trained as trainers to assist the Whole Child Program Director to continue the work after the grant ends.<br><br>Saginaw ISD can continue with evaluation after the grant ends-may be different than U of M

The Hexagon Tool: An Exploration Tool **Please create a copy and rename.**
Resource: University of North Carolina
Notes Page

have spent the past year with more time spent at home, cancelation of sports, reduced or eliminated PE time and restricted movement during the school day when in session (lunches in classrooms, specials teachers coming to the classroom to instruct). The need is even greater now than when we first visited the program on March 11, 2020… 2 DAYS before schools closed for the remainder of the school year.<br><br>Experts are estimating that we will see obesity rates rise due to COVID restrictions.<br><br>School staff are noticing that students need to move more but Michigan winter months make it challenging to get students outside regularly.<br><br>PE and recess options are restricted due to COVID safety precautions.<br><br>Teachers are wanting resources and support for incorporating physical activity while in the classroom that still keeps kids safe.<br><br>In the previous year, students were getting 1-2 days of PE a week. Now that number has reduced even when back face to face. This is due to the hybrid schedule where students are in person 2 days a week. It is also do to the “specials” teachers (PE, Music, Art, languages, etc) are subbing in classrooms when districts cannot get subs. This means that those classes do not take place during the day.	active and this allows youth to be physically active during school hours<br>• Schools are already implementing PA initiatives like GoNoodle and Just Dance activities in the classroom<br>• InPACT at School would be included as a part of Michigan Model training<br><br>**Concerns/Areas to be Intentional:**<br><br>• Teachers are in the process of simplifying Tier I instruction - would that be part of the training? PD District is already set. Sub costs? Release Time? (Covered by ISD)<br><br>• We don’t want this to be just one more thing. Not an add on.<br><br>• Fear of being methodical - Brain breaks are a place for autonomy. (Work with teachers on the importance of fitting them in when NEEDED, not always a set schedule)<br><br>• Fit with whole child - there are a lot of initiatives going on already. How do we blend this in seamlessly?<br><br>• Maybe have teachers do a time audit periodically to see how much time it takes.<br><br>• Fit with HBHM - extension	opportunities.<br>• Admin buy in (ISD, central, building)<br>• Buy in from PE teachers<br>• Buy in from Classroom teachers<br>• Buy in from 31n staff	Washtenaw, Wayne and Jackson (similar types of districts in these counties as compared to Saginaw ISD)<br><br>Other data schools would be interested in:<br><br>• Discipline data<br>• Attendance<br>• Academic Improvements	InPACT at School allows for adaptations for students. While the recommendation is for 4-5 minute breaks 4-5 times a day, that can be increased if needed based on the needs of the students that day. Also, students and teachers can select the types of movement they would like to do… the activities are not prescribed. The flexibility will be appealing to students and teachers alike.<br><br>**Pilot phase.**<br>**We are being offered to be part of the first replication.**<br><br>**Diverse settingings**<br>**Rural, Urban, Socio-Economic Status, Ethnicity**	Does not require ongoing costs, just time to implement.

Here are so other stats:<br> - Michigan is 5th worst state in US in obesity rates in 10-17 yr olds @ 18.9%. (State of Childhood Obesity) 48.6% of 7th grade students who took MiPHY stated that they are trying to lose weight. - Screen time is huge on 7th grade MiPHY data with over 54% students playing video/computer games 3+ hours a day and over 27% stating that they watch 3+ hours of tv on an average school day - PA drops by 75% from ages 9-15. <br>**Gaps Addressed:**<br><br>InPACT@School will provide school staff with best practices in integrating physical activity into the classroom schedule. This initiative will also help youth reach the recommended daily amount of time they should be physically active. Combined with PE and recess, students should be able to reach the 60 min recommendation with the 20 minutes (total) of InPACT at School activities.<br><br>School staff has also stated that students are fidgety in class, more so than in previous years and believe that this has to do with the pause in face-to-face learning for most of the school year.	(can HBHM's PA count that day?)				

The Hexagon Tool: An Exploration Tool **Please create a copy and rename.**

Resource: University of North Carolina

Notes Page
